# Integrative field scale phenotyping for investigating metabolic components of water stress within a vineyard

**DOI:** 10.1186/s13007-017-0241-z

**Published:** 2017-10-30

**Authors:** Jorge Gago, Alisdair R. Fernie, Zoran Nikoloski, Takayuki Tohge, Sebastiá Martorell, José Mariano Escalona, Miquel Ribas-Carbó, Jaume Flexas, Hipólito Medrano

**Affiliations:** 1 Research Group on Plant Biology under Mediterranean Conditions, Universitat de les Illes Balears, cta. de Valldemossa Km 7.5, 07122 Palma de Mallorca, Illes Balears, Spain; 2 Max-Planck-Institut für Molekulare Pflanzenphysiologie, 14476 Potsdam-Golm, Germany

**Keywords:** Drought, Metabolomics, Phenotyping, Remote sensing, Regression modeling, UAVs, *Vitis vinifera*

## Abstract

**Background:**

There is currently a high requirement for field phenotyping methodologies/technologies to determine quantitative traits related to crop yield and plant stress responses under field conditions.

**Methods:**

We employed an unmanned aerial vehicle equipped with a thermal camera as a high-throughput phenotyping platform to obtain canopy level data of the vines under three irrigation treatments. High-resolution imagery (< 2.5 cm/pixel) was employed to estimate the canopy conductance (*g*_*c*_) via the leaf energy balance model. In parallel, physiological stress measurements at leaf and stem level as well as leaf sampling for primary and secondary metabolome analysis were performed.

**Results:**

Aerial *g*_*c*_ correlated significantly with leaf stomatal conductance (*g*_*s*_) and stem sap flow, benchmarking the quality of our remote sensing technique. Metabolome profiles were subsequently linked with *g*_*c*_ and *g*_*s*_ via partial least square modelling. By this approach malate and flavonols, which have previously been implicated to play a role in stomatal function under controlled greenhouse conditions within model species, were demonstrated to also be relevant in field conditions.

**Conclusions:**

We propose an integrative methodology combining metabolomics, organ-level physiology and UAV-based remote sensing of the whole canopy responses to water stress within a vineyard. Finally, we discuss the general utility of this integrative methodology for broad field phenotyping.

**Electronic supplementary material:**

The online version of this article (doi:10.1186/s13007-017-0241-z) contains supplementary material, which is available to authorized users.

## Background

The challenging aims of precision agriculture and field phenotyping encompass the efficient use of resources whilst ensuring optimal crop yields [1, 2]. Currently, drought and other limitations of water availability represent some of the major threats for crop production in Mediterranean climates [1], where the grapevine is one of the most economical important crops that can drastically be affected by water scarcity in terms of both productivity and quality [3]. To attain the goals of field phenotyping a major shift in focus from leaf and whole plant physiology to the crop field is required. Such a shift necessitates the development of methods and technologies that can be integrative and readily up-scaled with the size of the system under study [4].

Recently developed unmanned aerial vehicles (UAVs) represent useful aerial platforms for such purposes. In contrast to other platforms, such as satellites and manned aircrafts, UAVs facilitate temporal and spatial remote sensing at adequate resolution when considering the highly dynamic relationship between vegetation and its environment [1, 2, 5]. Several pioneering works have demonstrated the usefulness of UAVs by revealing significant correlations between so-called plant-truth data recorded on the ground at leaf level with data from aerial remote sensing imagery and generate field stress maps useful for decision-making under the precision agriculture framework [6, 7]. This technology offers new opportunities for precision agriculture management due to the fact that the existing solutions for conventional measurements are time-consuming and of high cost, even in the case when a single canopy is investigated [8]; however the use of this technology beyond mapping stress in the crop fields to up-scaling from molecular mechanisms to whole plant responses are far from being frequent, most likely due to the high technological and multi-disciplinarity demands imposed by such studies.

The fact that drought has been recognized as a limiting factor for crop productivity worldwide has challenged the scientific community to develop markers that act as proxy for water stress estimators in crops. For this purpose, stomatal conductance (*g*_*s*_) has been proposed as a physiological parameter which reliably reflects the physiological status of several species including grapevine [3]. In addition, *g*_*s*_ is important due to the well-studied relationship between leaf temperature and transpiration, since this provides the opportunity of remotely estimating it from aerial thermography using the energy balance model [1, 5]. In parallel to these developments, technological advances in diverse metabolite profiling approaches have propelled a deeper understanding of the molecular mechanisms driving plant-environment responses at the molecular level as well as of metabolic phenotypic diversity and its underlying genetic variation [9–12]. Thus, integrative methodologies linking metabolism, physiological and agronomical traits from rapid and accurate phenotyping platforms with metabolite networks should prove invaluable in determining strategies for genetic improvement via reverse genetics or classical breeding [13].

Here, we introduce a novel integrative suite of methods to study the water stress under agricultural conditions using an UAV to obtain data response at canopy level linked through mathematical modelling with metabolite profiles of samples harvested from the same material. The multi-copter UAV was equipped with a thermal camera to obtain aerial thermal high-resolution images. These images were employed to estimate canopy conductance (*g*_*c*_) via a leaf energy balance model and correlated with more standardized traditional measurements including leaf water potential (*Ψ*), stomatal conductance (*g*_*s*_) and stem sap flow fluxes. They were subsequently linked to gas-chromatography (GC) and liquid-chromatography (LC) coupled to mass spec (MS) based metabolic profiling in three water stress treatments in our experimental vineyard. We believe that such integrative approaches could be highly useful as high-throughput phenotyping platforms for crop-field conditions considering the plant/community response to a changing environment across scales.

## Results

### Assessing vine physiological status canopy, stem and leaf levels

**Fig. 1:**
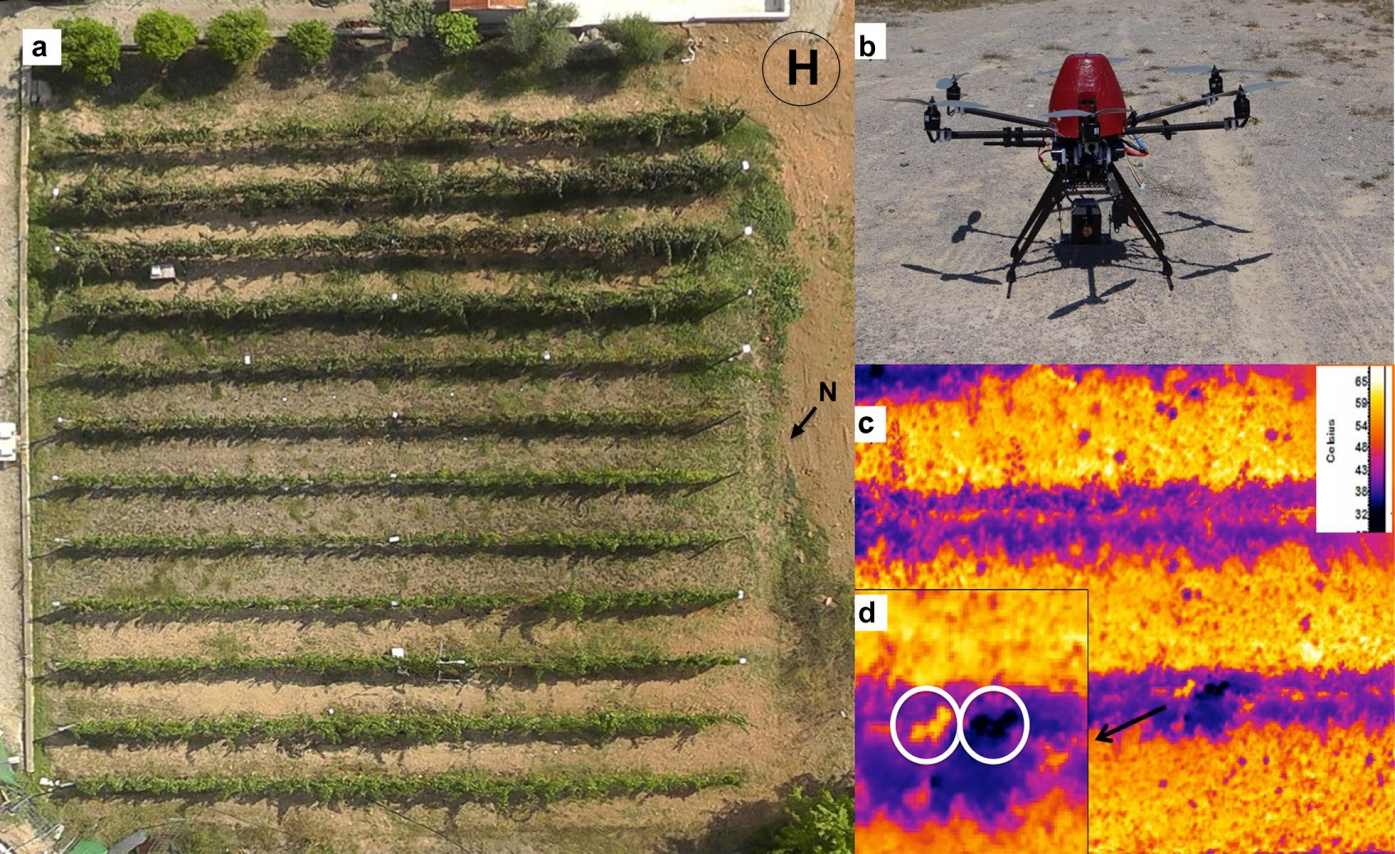
**a** RGB aerial ortho-mosaic of the experimental vineyard located at University of Balearic Islands (Mallorca, Spain). **b** UAV six engine multi-copter type carbon frame Air-Sci (UAVEurope, Spain) equipped with an uncooled thermal camera (Gobi384, Xenics, Belgium). **c** High-resolution aerial thermal picture (2.5 cm/px) of the vineyard and detailed view (**d**) of the dry and wet artificial references placed in the vine (inside white circles)

**Fig. 2:**
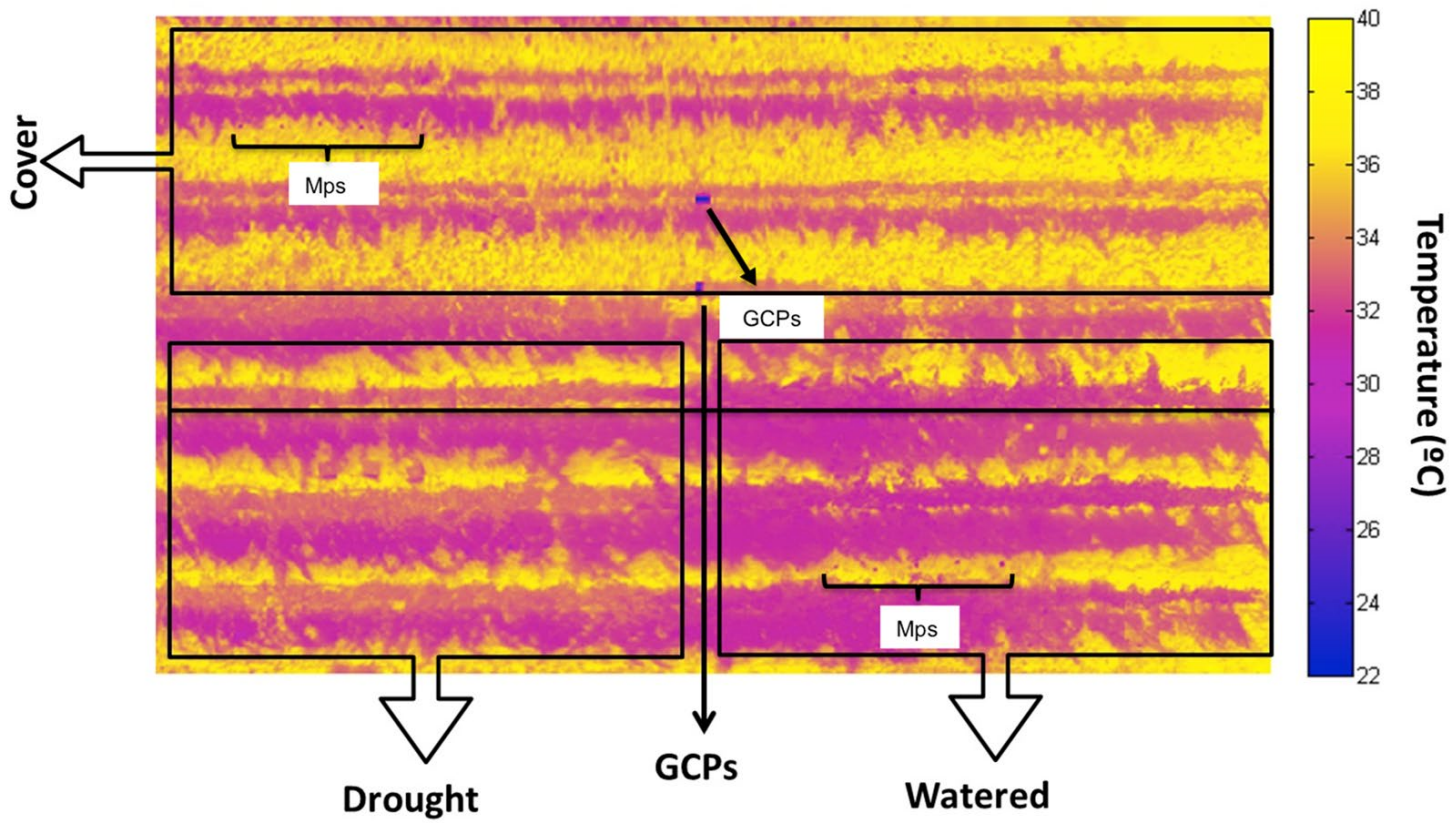
Aerial thermal imagery from 15 m above the vineyard at midday 29th August 2012. The high spatial resolution (2.5 cm/px) allowed extraction of pure canopy information. The different water treatments can be easily distinguished, cover-crop “C” at the top of the thermal mosaic, drought “D” bottom-left and the watered plants “W” in the bottom-right side. Watered plants can be visualized by the dark colors representing lower temperature meanwhile water stress treatments were brighter indicating higher temperature. Some ground control points (GCPs) employed for geo-referencing purposes are also visible in the middle of the vineyard. Temperature is expressed in celsius degrees (°C)

Currently, there is an essential and urgent requirement to integrate molecular, physiology and field phenotyping techniques to explore quantitative traits related to productivity and stress response beyond lab experiments. In this work, we selected to impose three different irrigation treatments in our experimental vineyard (Fig. 1a). Subsequently, employing an UAV multi-copter type equipped with a thermal camera (Fig. 1b) we obtained high-resolution thermal imagery (< 2.5 cm/px) (Figs. 1c, d, 2) to determine at canopy level the water stress of the different treatments through the thermal indices and the canopy conductance calculated from the leaf energy balance. In parallel, we determined the stem sap flow fluxes, gas-exchange measurements at leaf level and sampling the very same leaves to determine the primary and secondary metabolic profile annotating 60 metabolites. Finally, further modelling using the partial least square (PLS) technique allowed us to link the metabolic profile with plant stress responses at leaf and canopy levels.

The physiological status of the vines under the three different irrigation regimes is presented in Table 1, with all measurements of gas-exchange and sap flow collected at the same time as the UAV flight. We investigated three scenarios: two irrigation deficient treatments: cover-crop (C) and drought (D), as well as watered plants as control treatment (denoted by W) in our experimental vineyard (Fig. 1a) (see Experimental procedures). We observed significant reductions in photosynthesis, transpiration and stomatal conductance measured at the leaf level with the infrared gas-analyzer for both deficit irrigation treatments. Similarly, the leaf water potential was significant lower for both water stress treatments at pre-dawn and midday measurements compared with the W treatment. Sap flow fluxes displayed a similar trend, with a significantly lower stem sap flux in D vines (0.01 l m^−2^) than in W vines (0.04 l m^−2^).

**Table 1:** Vines physiological status in the irrigation treatments

Treatment	A (µmol CO_2_ m^−2^ s^−1^)	E (mmol H_2_O m^−2^ s^−1^)	g_sw_ (mol m^−2^ s^−1^)	Sap flow (l min^−1^ m^−2^)	Ψ_pd_ (MP_a_)	Ψ_md_ (MP_a_)
W	12.26 ± 2.2 a	7.44 ± 1.2 a	0.15 ± 0.037 a	0.04 ± 0.024 a	− 0.24 ± 0.06a	− 1.00 ± 0.04a
D	2.24 ± 0.6 b	1.27 ± 0.26 c	0.02 ± 0.003 b	0.01 ± 0.005 b	− 0.85 ± 0.06b	− 1.88 ± 0.03b
C	3.66 ± 1.9 b	2.54 ± 0.42 b	0.04 ± 0.007 b		− 0.71 ± 0.08b	

Gas-exchange and hydric status of the vines in our experimental vineyard under three different irrigation treatments (W is well-watered, D is drought and C is cover crop), mean values ± SE are shown of net CO_2_ assimilation (*A*), transpiration (*E*), stomatal conductance of water vapour (*g*_*sw*_) at leaf level, and sap flow fluxes and pre-dawn and midday water potential (*Ψ*_*pd*_ and *Ψ*_*md*_, respectively) at stem level. Different letters denote significant differences by multiple’s comparisons Tukey’s test (p < 0.05, *n* = 6)

Canopy temperature differences between treatments were also observed using the multi-copter UAV equipped with a thermal camera (Fig. 1b), imagery resolution allows us to distinguish leaf references placed in the vine canopy to calibrate the thermal indices (Fig. 1b, c), the aerial thermal mosaic image composition obtained from 15 m above the vineyard can be seen in Fig. 2. Multi-copter technology allowed us to fly very close to the ground and thus obtain high-thermal resolution (2.5 cm/px), further enabling the collection of pure canopy pixels avoiding interfering noise from the background/soil (Figs. 1b, c, 2). Watered plants can be clearly observed to display lower temperature (dark blue color) whilst C and D treatments showed higher temperatures (bright purple color). It is important to note that both C and D treatments resulted in considerably reduced leaf area. In watered plants (W) total leaf area ranged from 4 to 5.3 m^2^ while in C and D treatments this was lower than 2.5 and 3.5 m^2^, respectively (Fig. 2).

Dry and wet artificial leaf references and the canopy temperature extracted from each plant (Fig. 1c and 1d) were next used to calculate the thermal indices CWSI, IG, I3 and T_c_ − T_a_. These thermal indices were developed previously as a simple approach to estimate the water status based in the cooling effect performed by plant transpiration, dry and wet artificial references show the minimum and maximum hypothetical transpiration, thus maximum and minimum temperature, respectively, under the current meteorological conditions (see “Methods” section for a full description of how we calculated these indices). In our case, leaf artificial references were placed in the field that were similar in shape and size to vine leaves and placed in the vine canopy to obtain temperature references closer to the micro-meteorological environment of the vines, a fact that was possible due to the high-thermal resolution which was sufficient to identify these artificial references amongst the canopy (Fig. 1c, d). The relationship between these indices and *g*_*s*_ and sap flow is shown in Additional file 1: Fig. S1. As previously described CWSI and I3 showed an inverse relationship with *g*_*s*_, whilst IG showed a positive relationship [14, 15]. Significant functions were observed between all thermal indices and leaf stomatal conductance (R^2^ 0.78–0.86) and stem sap flow (R^2^ 0.69–0.76), respectively.

**Fig. 3:**
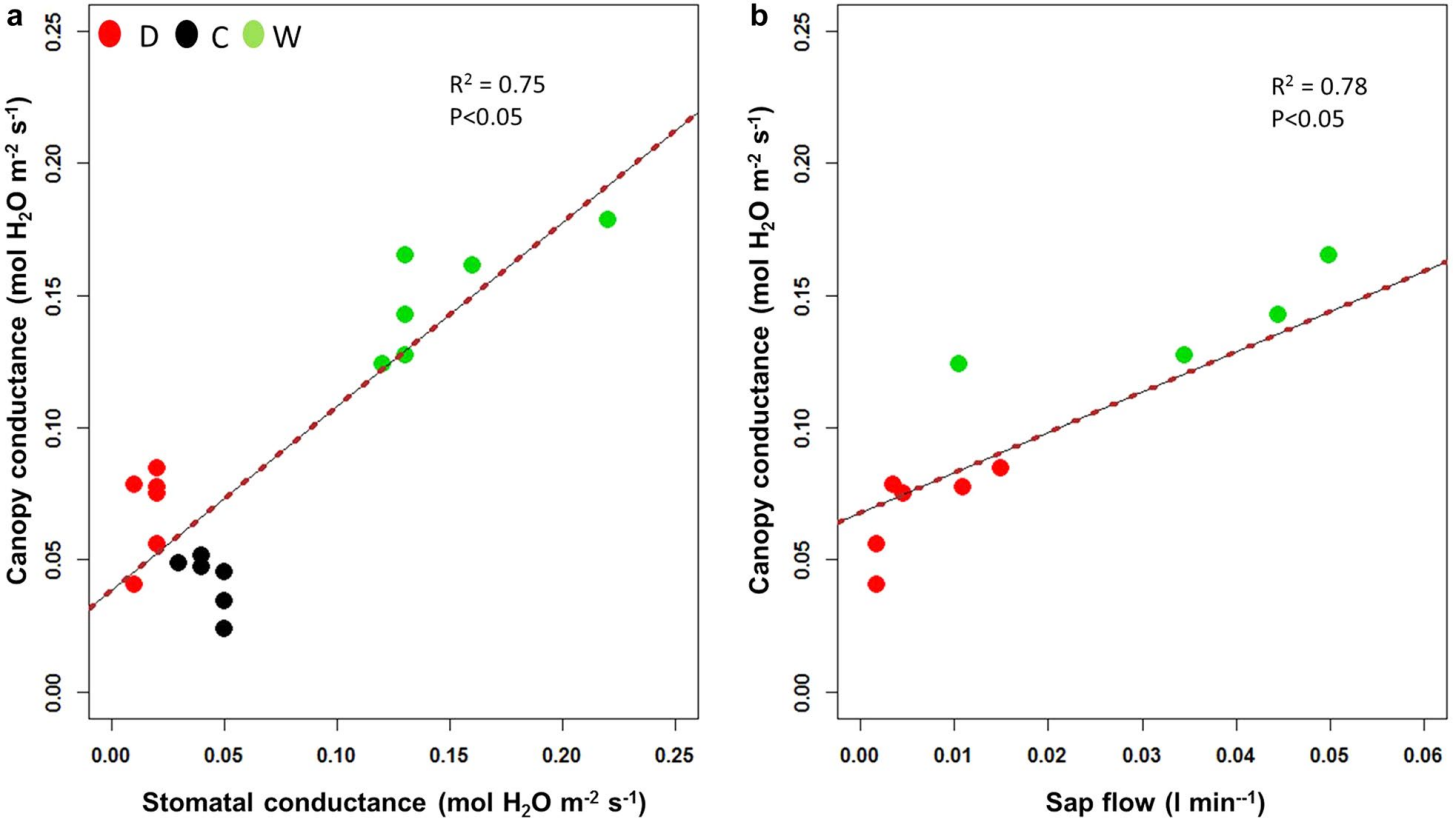
Relationships obtained between the canopy conductance (g_c_) estimated from aerial thermal imagery using the leaf energy balance model (Jones [15]) and stomatal conductance (g_s_) measured in leaves (**a**, *n* = 18) and stem sap flow fluxes (**b**, *n* = 10) (p < 0.05) at noon 29th August 2012. Green, red and black dots correspond to watered (W), cover-crop (C) and drought (D) irrigation treatments

Next we estimated canopy conductance (*g*_*c*_) using the canopy temperature obtained from the UAV combined with meteorological data via the leaf energy balance model [15, 16]. Figure 3 shows the relation between the estimated *g*_*c*_ and the *g*_*s*_ measured at leaf level with a gas-exchange infrared analyzer, and as with the previous thermal indices, we observed a significant relationship between canopy conductance and stomatal conductance (R^2^ = 0.75) and also for sap flow measurements (R^2^ = 0.78). Furthermore, as *g*_*s*_ was measured by the gas-exchange analyzer at leaf level and *g*_*c*_ was estimated at canopy level (and both calculations depend of the temperature), we observed that temperature measured by both techniques were in the same range and no significant differences were found that will affect the calculations of either parameters or the differences between them (Additional file 1: Figure S2).

### Leaf metabolite profiles following water stress

A total of 60 metabolites were identified and quantified in independent GC–MS and LC–MS analyses from fully-expanded leaves for each of the three irrigation treatments.

**Fig. 4:**
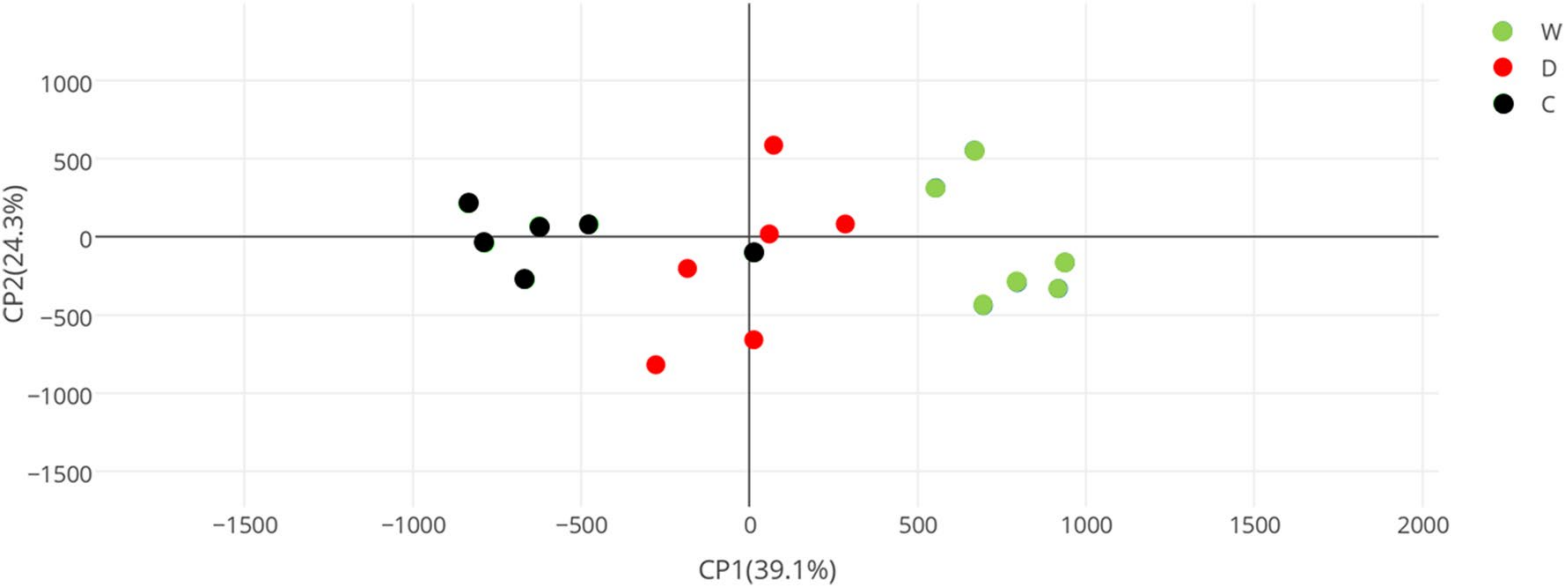
PCA analysis of metabolite profiles of fully-expanded leaves harvested at noon 29th August 2012 from grapevine cv. Grenache under the three different irrigation treatments (*n* = 18). Green, red and black dots correspond to watered (W), cover-crop (C) and drought (D) irrigation treatments

A general overview of the metabolomics profiles of the treatments was realized using a principal component analysis (PCA) (Fig. 4). PCA determined five components which in total explain 95% of the total variation, the first two components jointly explain 63.4% of the variance. The principal component 1 explains 39.1% of the variation, and along this major component watered (W) treatment and the two irrigation deficient treatments (D and C) are separated.

In this experiment, 46 and 41% of the measured metabolites displayed significantly different levels between the control and the water deficient irrigation treatments C and D, respectively (Additional file 1: Table S1). Some metabolic traits displayed consistent changes in both water stress conditions. Specifically, 19 and 20 metabolites increased meanwhile nine and five decreased significantly, in the C and D treatments, respectively. In addition, eight of those which displayed increases and five which showed decreases were common to both treatments (Additional file 1: Table S1). It is important to note that the metabolite data was expressed on a sample dry matter basis and that differences between treatments are, therefore, unrelated to the large changes in the volume of cellular water between the treatments.

**Fig. 5:**
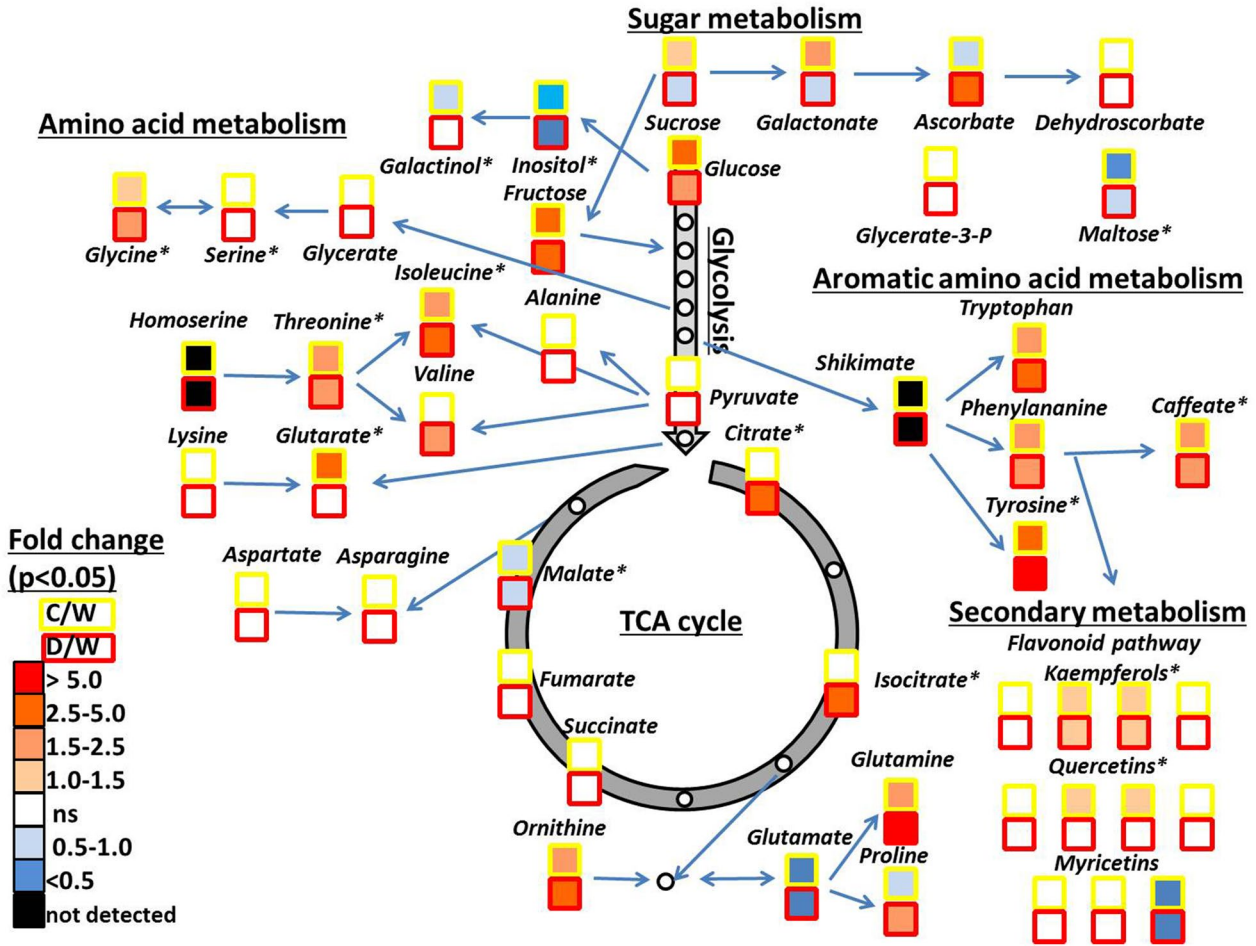
Metabolic pathways showing major alterations of vines imposed to deficit irrigation treatments. Color scale indicates the metabolite significant log^2^ fold-change (Tukey’s test α < 0.05, *n* = 6) of each treatment with respect to watered plants (W), and the deficit irrigation treatments C (cover-crop) and D (drought) treatments are signaled by the square frames yellow and red, respectively. Significant metabolites observed by PLS modelling for g_c_ and g_s_ are signaled by an asterisk (p < 0.05)

Figure 5 displays metabolic pathway mapping of the major metabolic alterations due to water stress imposed on vines. Many amino acids increase up to 2.8-fold in comparison with the control condition; these include: valine, isoleucine, threonine, asparagine, and the precursors of polyamines (e.g., putrescine and spermine), i.e., ornithine, tryptophan and glutamine. Proline significantly increased in the treatment D but decreased in treatment C. The sugar-alcohols *myo*-inositol and galactinol decreased upon both irrigation deficient treatments.

With the exception of citrate, which increases sharply in the D treatment, most other organic acids related to the tricarboxylic acid (TCA) cycle, such as pyruvate, succinate, and fumarate are at similar levels in all conditions. However, malate displayed significantly decreased contents. By contrast, the major hexoses, glucose, fructose, as well as altrose, showed considerable increases in the water deficient treatment, whilst, ribose and maltose decreased. Conversely, sucrose was reduced in leaves subject to treatment D, but accumulated in treatment C. Caffeate displayed considerable significant increases in both water deficient treatments, however accumulation of the major antioxidant ascorbate was only observed for treatment D (Fig. 5). Changes in the levels of ascorbate precursors such as galactonic acid 1-4 lactone, galacturonic and glucuronic acid were, however, only increased in treatment C following the same trend as for sucrose which is likely their precusor. Subsequently, we also checked flavonol/phenylpropanoid levels by LC–MS. Significant increases between the irrigation deficient treatments and the watered plants were observed in only 45% of metabolites of this class of compounds. That said, some specific compounds such as quercetin-3-*O*-glucuronide/glucoside and kaempferol-3-*O*-galactoside/glucoside displayed significantly higher relative content (Additional file 1: Table S1).

### Linking metabolome with leaf and canopy conductances through partial least square modelling

We next investigated the relationship between the drone measurements for water stress assessment obtained at canopy level as the *g*_*c*_ and also at leaf level as *g*_*s*_ with the leaf metabolic traits through Partial Least Square (PLS) regression modelling to improve our understanding under real field water deficit conditions. We determined PLS regression models using all measured metabolites as independent variables and *g*_*c*_ measured by the drone and *g*_*s*_ at leaf level as two individual dependent variables (i.e., responses). The models were determined by using the metabolic profiles as predictor variables which were scaled and centered.

The PLS regression determines models for each of the two responses, namely, *g*_*c*_ and *g*_*s*_, based on a certain number of components used. In the following, we focus on ranking and further interpreting the models which are of high predictive power. The latter can be quantified by the (root) mean squared error of prediction (R)MSEP and coefficient of multiple determination (R^2^), bias-corrected via cross-validation.

Inspection of the RMSEP indicated that the smallest values, of 0.028 and 0.039, for the models of *g*_*c*_ and *g*_*s*_ was obtained when using two and four components, respectively (Additional file 1: Fig. S3). The R^2^ coefficient for the respective number of components was 60.71 and 57.66% for *g*_*c*_ and *g*_*s*_, respectively (Additional file 1: Fig. S4). Therefore, we next inspected the coefficients of the PLS model with two components for *g*_*c*_ and four components for *g*_*s*_.

The coefficients of the models for *g*_*c*_ and *g*_*s*_ show positive correlation 0.703, *p* value < 0.001, as can be seen from the concordance of the model coefficients with respect to the two components (Additional file 1: Fig. S5). The largest positive coefficients for *g*_*c*_ were observed for: phosphorate, malate, isomaltose, butyric acid, ribose, galactinol, and *myo*-inositol, while the largest negative coefficients were for quercetin-3-*O*-galactoside, maleate, kaempferol-3-*O*-galactoside, kaempferol-3-O-glucoside, myricetin-3-*O*-glucuronide, and asparagine. Interestingly, 39 of the coefficients were negative and 21 positive. The largest positive coefficients for *g*_*s*_ were observed for: ribose, malate, butyric acid, lysine, and maltose, while the largest negative coefficients were for: serine, citrate, quercetin-3-*O*-galactoside, kaempferol-3-*O*-glucoside, kaempferol-3-*O*-galactoside, and myricetin-3-*O*-glucuronide. For this trait, 31 of the coefficients were negative and 29 positive.

To rank the importance of metabolites in the determined models of *g*_*c*_ and *g*_*s*_, respectively, we calculated their corresponding variable importance in projection (VIP) values. To this end, we report all variables which have VIP values higher than the average in the respective model. In the PLS model for *g*_*c*_, the 15 top-ranked metabolites based on the VIP are (from highest to lowest): quercetin-3-*O*-galactoside, malate, kaempferol-3-*O*-galactoside, kaempferol-3-*O*-glucoside, caffeate, *myo*-inositol, maltose, 2-oxo-gulonic acid, galactinol, glucose, glutarate, maleate, altrose, and glucuronic acid. In the PLS model for *g*_*s*_, the 15 top-ranked metabolites based on the VIP are (from highest to lowest): serine, kaempferol-3-*O*-glucoside, malate, kaempferol-3-*O*-galactoside, isoleucine, citrate, tyrosine, threonine, quercetin-3-*O*-galactoside, isocitrate, caffeate, *myo*-inositol, erythronic acid and glycine.

**Fig. 6:**
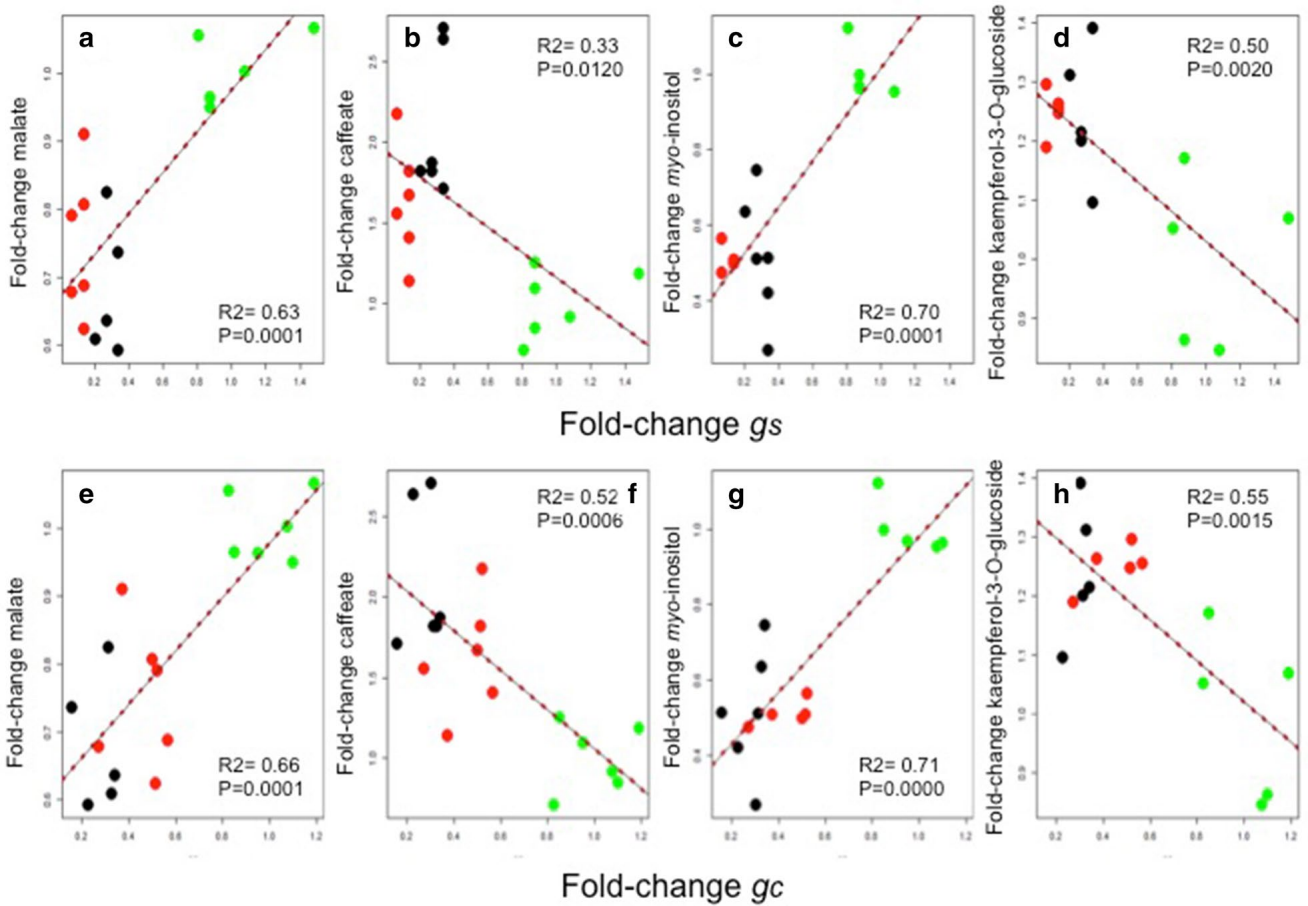
Relationships obtained between the stomatal conductance (*g*_*s*_) measured in leaves (**a**, *n* = 18) and the canopy conductance (*g*_*c*_) estimated from aerial thermal imagery using the leaf energy balance model (Jones [15]) with the metabolites malate (**a**, **e**), caffeate (**b**, **f**), myo-inositol (**c**, **g**) and the flavonol kaempferol-3-*O*-glucoside (**d**, **h**). Green, red and black dots correspond to watered (W), cover-crop (C) and drought (D) irrigation treatments

Almost half of these metabolites appear to have high VIP values in the models for both *g*_*c*_ and *g*_*s*_; these metabolites include all the flavonol/phenylpropanoid metabolites selected based on the VIP values as well as malate, caffeate and *myo*-inositol. In the Fig. 6 are shown the direct significant relationships between these metabolites and *g*_*s*_ at leaf level (R^2^ 0.33–0.70) and *g*_*c*_ at canopy level estimated using the UAV (R^2^ 0.55–0.71). In addition, it is important to note that although amino acids appeared to be important for *g*_*s*_, this was not the case for *g*_*c*_. A contrary pattern was observed for the group of sugars, whereby none was listed to have high VIP value for *g*_*s*_ but this compound class provided 20% of the metabolites deemed of high VIP value for *g*_*c*_. Diverse organic acids complete the list of metabolites important for the modelling of both traits.

## Discussion

Several authors have suggested that there is a lack of concordance between results obtained in highly controlled growth chambers and those under field conditions [2]. As such there is currently great interest in developing better approaches towards phenotyping within the field [2, 17]. Thus, one of the major challenges of plant phenotyping is the acquisition of high-throughput field data that finally can be integrated with other useful information provided by other post-genomic technologies to decipher phenotypic diversity and its underlying genetic variation [2, 18].

In this work, the flight capacities of multi-copter type UAVs allow them to fly closer to the ground thus improving importantly thermal resolution [5, 19–22]. The high-thermal resolution images obtained (2.5 cm/pixel obtained at 15 m height, Figs. 1b, c, 2) improves on previous thermal imagery from other type of UAVs such as helicopters and wing-span type vehicles (12–49 cm/pixel obtained at 150–575 m height) [20] as well recently published data with UAVs multi-copter type (9–13 cm/px) [21, 22]. They additionally lack land-off requirements (in the Fig. 1a the landing point employed close to the vineyard can be seen), and can easily hover (i.e. maintain highly stable in-flight positions), as well as being able to perform low-speed flights at low altitude. The combination of these factors thus renders such multi-copters highly useful for several applications for precision agriculture and phenotyping programs [20].

In this sense, *g*_*c*_ estimation from aerial data and thermal indices showed significant relationships (p < 0.05) with *g*_*s*_ and sap flow ensuring the reliability of these indices as proxies of water stress in our experimental conditions (Fig. 3, Additional file 1: Fig. S2) [6, 8, 21–23]. Interesting, all previous relationships concerning aerial thermal data from canopy level were restricted to physiological parameters from leaf level such as *g*_*s*_ and Ψ, but still no information is available with stem sap flow fluxes that can be considered a more integrative approach for the general plant status [24].

On the other hand, previous studies have demonstrated that water stress significantly affects the leaf metabolome [9, 25–27] and qualitative and quantitative variation of metabolites have traditionally been linked to both genetic factors through mapping approaches as well as to morphological traits such as biomass [17, 28, 29]. Still currently, no information is available regarding the relationships between canopy and stomatal conductances with primary and intermediate metabolism, however both traits are essential for agricultural water use efficiency and for the predicted climatic change for the Mediterranean and semi-arid regions. In the current work we collected leaf samples for metabolomics in parallel with the aerial remote sensing UAV campaign plus conventional physiological measurements as plant-truth data, approximately 45% of the measured metabolite levels were significantly affected on water-deprivation (Fig. 5; Additional file 1: Table S1). Interestingly, PLS modelling revealed significant relationships with *g*_*c*_ and *g*_*s*_ with different metabolites related to antioxidant activity and osmotic potential which can ultimately affect guard cell responses and as such water fluxes.

Some of the most significant metabolite increases in the irrigation deficient vine treatments were related to anti-oxidant compounds for example, ascorbate (and precursors of ascorbate such as the organic acids galactonic, 1-4 lactone, galacturonic and glucuronic acid), citrate, *trans*-caffeate and flavonols/phenylpropanoids (and their precursors such as Phe, Try and Tyr) (Figs. 5, 6). Accumulation of reactive oxygen species (ROS) provokes oxidation and dysfunction of cellular components, thus ensuring the reducing power for the maintenance of the cell redox state via the operation of ROS scavenging mechanisms is an important task under conditions of stress [9]. Moreover, flavonols/phenylpropanoids can play a role related to thermal and excess energy dissipation and photoprotection [10, 30]. *Trans*-caffeate is a precursor of secondary metabolism, and several secondary metabolites such as kaempferol and quercetin-derivatives of the flavonol/phenylpropanoid family have previously been reported as dampeners of ABA oxidative stress response to stomata guard cells movements [30].

The modelling approach also revealed several metabolites with a known osmolyte role such as proline, as well as sugars including fructose, glucose and sucrose [25, 27]. Previously, in other woody plants such as *Eucalyptus* and *Acacia* it was described that fructose, sucrose and glucose as well sugar-alcohols, proline and GABA make major contributions to osmotic adjustment [25]. Rizhsky and co-authors (2004) [31] observed that in *Arabidopsis* when defense pathways collide to response to different stresses such as heat and drought (as actually can be observed in crop fields in semi-arid regions), sucrose is mostly accumulated instead of proline as the major osmoprotectant given that the toxic effect of the amino acid is enhanced under heat stresses. In grapevine, previous comparisons of leaf metabolic profiles of two different cultivars growing both in a semi-arid and temperate regions in the West Bank in Palestine, showed that both cultivars in the semi-arid region showed higher levels of amino acids as Val, Iso, Asp, Lys, Met, Thr, Trp, Tyr, Ser and Pro, and as well organic acids such as citric and isocitric acids, whilst levels of major sugars as Glc, Suc and Gal remain quite stable for both cultivars between both regions [32]. Secondary metabolism from the flavonols family (i.e. myricetin, quercetin and its derivatives, and kaempferol) was also studied and as previously observed for sugars no important alterations were observed for secondary metabolites between locations. These results suggest that the acclimation to semi-arid regions are mediated by amino acids either as solutes and osmotic adjusters to maintain the cell homeostasis or to be mobilized for the development of new proteins for plant stress tolerance (Fig. 5). However, it can be anticipated that plants are acclimated to the culture conditions in each zone and probably do not suffer relevant stress and this could, therefore, be the reason that authors did not find changes in sugars and secondary metabolites [32]. From a viticulturist point of view, water stress could be an interesting ally to improve the quality of the berries, several studies pointed out that increase the synthesis and accumulation of flavonols and other polyphenols and carotenoids that will finally increase pigmentation, flavor and promote fruit well-known health benefits as well the economic value of production [33, 34].

Here, we also observed significant accumulation of sugars for water stress treatments, suggesting a possible role of these compounds in osmotic cell adjustment under combined stresses in grapevine. Recent, studies have begun to dissect the complex relationship and key-roles between stomatal response and photosynthesis and respiration metabolisms [35–37]. Sugar production and transport from the mesophyll to guard cells is a key point regulating the tight relationship between *A* and *g*_*s*_ [38, 39]; accumulation of sugars in the guard cell apoplast space by the transpiration stream induces stomatal closure (the process is still not well understood), also, sugars can enter guard cells and provoke stomatal closure through abscisic acid (ABA)-dependent mechanism mediated by hexokinases [38, 40, 41]. Malate, the organic anion driving guard cell turgor, is also an organic acid intermediate of the TCA cycle, and was one of the most significant metabolites related to *g*_*c*_ and *g*_*s*_ (Figs. 5, 6). The role of malate as an organic anion regulating stomata cell guard turgor, and thus promoting changes into stomatal aperture and conductance is long established [42]. However, as stated by the authors of this study guard cell movements mediated via malate will additionally be regulated by other well-characterized mechanisms (K^+^, Cl^−^, Ca^2+^ and ABA) [43]. It thus seems likely that the relative importance and molecular hierarchy of these regulatory processes that will probably vary with the prevailing conditions. Moreover, in the Mediterranean summer vines are exposed to elevated temperatures and solar radiation, thus our experimental conditions not only imposed water scarcity but also heat and light stress on the plants. Currently, combined stress and/or field experiments describing metabolomic responses still remain scarce in the literature. Whilst it is true that under lab conditions thousands of data from the most important crops species under different type of stresses are being produced, extrapolating this knowledge to real crop field conditions is still an important challenge for plant scientists and industry; the requirement of common physiological status indicators [44], anatomical traits [45, 46] and/or normalized metabolic markers [9] would be highly desired to facilitate integrative modeling strategies and mining knowledge from extensive experiments. This means that mutiple overlapping measurements in lab and field will be needed to ensure a similar physiological and metabolic status in several traits prior to cross data comparison from different sources. This statement notwithstanding, a fuller comprehension of metabolic and physiological mechanisms in an integrated manner, together with fast and accurate field phenotyping methods, will likely great aid in facilitating engineering of sustainable increases in crop yield during the present change in climatic conditions.

## Conclusions

Here, we present an integrative methodology combining metabolomics, leaf-organ physiology as well whole-plant data using an UAV-based remote sensing platform as a rapid and accurate high-throughput phenotyping platform for in field work. These methodologies/technologies can promote a better understanding of the complex interaction between plant and environment under water deficit using the *g*_*s*_ (leaf level) and *g*_*c*_ (canopy level) responses in a Mediterranean experimental vineyard as a case study. Using mathematical modelling we were able to identify metabolites whose levels most closely corresponded to the observed physiological alterations whilst several of the candidate metabolites are well described in the literature as corresponding to similar changes in controlled growth chambers; this study represents their first confirmation in crop species under real field conditions. We therefore believe that this approach will prove a highly effective phenotyping approach and are currently exploring possibilities to improve our knowledge in spatial and temporal biological resolution across scales.

## Methods

### Experimental site and environmental conditions

The study was carried out in an experimental vineyard located in the University of Balearic Islands (Mallorca, Spain) the 29th of August 2012 (Fig. 1a). Four year old *Vitis vinifera* L. “Grenache” cultivar plants grafted on Ritcher-110 were employed for this work. Vines are planted in rows (2.5 m between rows) and spaced 1 m between each plant orientated N–NE to S–SW. Three irrigation treatments were applied: well-watered (W, 50% ET_o_) and two deficit irrigation treatments: cover crop (C) and drought (D) (Fig. 2). Cover crop treatment consists in a strip of herbaceous vegetation (20 × 10 m) established in the inter-rows (no tillage), with a permanent resident vegetation formed by a mixture of graminoids and leguminous plants, mostly *Medicago trunculata*, *Medicago polymorpha*, *Lotus ornithopo*-*dioides*, *Trifolium scabrum*, *Hordeum* sp. and *Chrysanthemum coronarium*. Grass cover area between the vine rows was controlled by manual practices to be homogeneity, cover crop is applied during all the spring (March to May) and at the beginning of the summer, mid of June herbs are cutted and no tillage is performed. Cover crop is a well-known agricultural practice commonly employed to reduce erosion by water runoff, improve fertility and soil structure in the crop fields as well to favour the competition between the herbaceous community and the crops to extract excessive water and nutrients within the effective root zone of plants, which can induce excessive vigor in grapevines. Currently, this technique is becoming increased interests for vineyards under Mediterranean environmental conditions, to optimize the season period as well the species employed to finally reduce the vine foliar area, and thus reducing the total transpiration during the summer period to increase the water use efficiency and crop quality [47]. In the drought treatment water was applied (50% ET_o_, as in the W treatment) up to the beginning of June, from this moment no irrigation was applied during the rest of the summer.

A research meteorological station was placed at the vineyard experimental site (Meteodata 3000, Geonica, Spain). Air temperature and relative humidity (STH5031), total solar radiation (pyranometer LiCOR LI-20SZ, USA), photosynthetic active radiation (Quantum sensor Li-COR Li-190R, USA), wind speed anemometer (anemometer Young 03002, USA). Aditionally, canopy temperature (Campbell thermal camera IR-120, 20° FOV) was also logged using a Campbell CR1000 cataloguer, sensor was installed 0.5 m vertically downward (nadir view) obtaining 0.36 m pure diameter canopy spot measurement that was employed to validate with ground-truth measurements the aerial thermal measurements acquired from the UAV.

Environmental conditions at this site are typical for a Mediterranean summer, with elevated temperatures along the day (mean 26.80 °C, and maximum temperatures of 37.3 °C), high irradiances ranging from 250 to 350 sunshine hours per month with considerable peaks of Photosynthetically Active Radiation (PAR) > 1500 μmol m^−2^ s^−1^, almost no precipitation, mean relative humidity of 55.57% and with elevated values of evapotranspiration (ETP, 130–160 l m^−2^ per month). Meteorological conditions during the UAV flight at noon of 29th August 2012 were within these ranges with mean values of 31.4 °C and 41.7% of air temperature and humidity relative (HR), respectively, with almost no wind (0.91 m/s) and short-wave radiation (SWR) of 659.5 W/m^2^.

### Plant physiological measurements

Leaf gas-exchange, photosynthesis (*A*) and stomatal conductance (*g*_*s*_) were measured at noon (coordinated with UAV remote sensing measurements see below) under clear sky and saturating light conditions in young, totally cenital exposed and fully-expanded leaves, typically between sixth to eighth from the apex as described previously [48], located the stem in a central position in the south face of the canopy of six plants per treatment using a Li-Cor 6400 infrared gas analyzer open system (IRGA; Li-6400, Li-Cor, Inc., Lincoln, NE, USA). A 2 × 3 cm standard leaf chamber model was employed using the natural sun radiation. CO_2_ concentration was set at 400 μmol CO_2_ mol^−1^ air with an air flow rate of 300 μmol s^−1^ meanwhile chamber temperature was unregulated (measurements were performed in under 3 min), and vapor pressure deficit (VPD) at these conditions was ca. 5.8 ± 0.2 kPa.

Plant water status was estimated by pre-dawn and midday leaf water potential measurements (*Ψ*_pd_ and *Ψ*_md_, respectively), using a Scholander pressure chamber (Soil moisture Equipment Corp. Santa Barbara, California USA). Leaves were sealed in a plastic bag, covered with aluminum foil. After 1 h, water potential was measured on one leaf per plant in four plants per treatment.

Sap flow was measured by the stem heat balance method using the standard Sap Flow meter P 4.1 from environmental measuring systems (EMS, Brno, Czech Republic), as described previously [49]. The mass flow of sap was estimated from the balance of heat fluxes up and down of the heated section of the stem [50, 51]. Sensors were insulated with 2 cm thick open-porous polyurethane foam and protected from radiation with aluminum foil. Gauges were installed in one representative stem per plant in four plants in the treatments W and D.

### Aerial remote sensing with a multi-copter type UAV

The UAV platform used in this study was a six-engine multi-copter (Mikrokopter^®^, Germany), diameter size 155 cm (without propellers), engines MK3538 and APC propellers 12 × 3.8 inches; under this configuration the aircraft have an estimated payload capacity of 2.2 kg (Fig. 1b). Autonomous flight through a pre-defined route was set using the onboard navigation system based on a GPS receiver (U-blox LEA6S) connected to a navigation board (Navy-Ctrl 2.0) and a small Microelectromechanical System (MEMS)-based IMU (Inertial Measurement Unit) (Mikrokopter Flight Controller ME V2.1), all the systems was powered by a Li–Po Battery 5A 30C, with this design and good weather conditions (reduced wind) flight time of the aircraft is ca. 22 min. The multi-copter has a stabilized camera mount to which a thermal camera Gobi384 (Xenics, Belgium) equipped with an 18 mm f/1 lens was attached (Fig. 1b). The image sensor is an uncooled microbolometer (a-Si) with a resolution of 384 × 288 pixels and a 25 × 25 μm pixel size. The camera also implemented non-uniformity correction (NUC) for internal calibration. The range of spectral response is 8–14 μm with a sensitivity of 0.05 K at 30 °C and it was radiometrically calibrated in lab conditions using a blackbody (SR-800R,CI Systems, USA). The field of view (FOV) is 25.5° which delivered high-thermal image resolution (ground pixel size) of 2.5 cm/pixel at typical established flight altitude of 15 m above the terrain. Additionally, empirical surface thermal calibrations were realized following the methodology proposed previously [5] with an accuracy better than 1 K.16-bit thermal images were acquired at 25 Hz from the camera by Ethernet and stored in the EPIA PICO-ITXE P710 board. With this extra additional weight (ca. 1 kg) flight time through the pre-defined autonomous way-point route over the experimental vineyard was set to 5 min to avoid low battery risks.

Emissivity (set to 0.96 within the range for leaves [52]), due to the low flight altitude radiometric distortions due to the atmospheric influence can be considered negligible and therefore no corrections were necessary [53]. Image post-processing was carried out using *Photoscan Professional* (Agisoft, Russia), a three-dimensional surface reconstruction based into the overlapping between pictures is generated providing high-density survey observations. The software performs an automated computed vision *Structure from Motion* (SfM) procedure implementing several steps like feature identification, matching and bundle adjustment, aligning the 16-bit imagery captured by the thermal camera. The dense 3D point is employed to generate a polygon mesh where the pixel values of the imagery are projected to develop an orthomosaic. Further details on the matching algorithms implemented in the software can be seen in several publications [54–56].

Ground control points (GCP’s) were used for geo-referencing of the images using this software. Several GCP’s were build covering a 20 × 10 cm steel plate with aluminium foil to ease its visualization in thermal images. Error was assessed using the root-mean-square error (RMSE) of GCP’s (ground control points) with values < 0.05 m. Aluminium foil marks was also used to define the different plants for each treatment and to help to distinguish precisely each target vine and the data extraction from the model. Finally, high-resolution digital terrain models (HR-DTM) and orthophotos are performed with this software.

### Thermal indices and leaf energy balance estimation

To estimate vineyard water stress different methodological approaches were used, as thermal indices: crop water stress index (CWSI) (Eq. 1) [14], stomatal conductance index (I_G_) (Eq. 2) and a reformulation of I_G_ (I_3_) (Eq. 3) [15].

1 $$CWSI = \frac{{T_{canopy} - T_{wet} }}{{T_{dry} - T_{wet} }}$$

2 $$IG = \frac{{T_{dry} - T_{canopy} }}{{T_{canopy} - T_{wet} }}$$

3 $$I3 = \frac{{T_{canopy} - T_{wet} }}{{T_{dry} - T_{canopy} }}$$

where *T*_*canopy*_ (°C) was the temperature extracted from the thermal images of the canopies of the vines, T_dry_ (°C) and T_wet_ (°C) are the temperature from the “reference surfaces” made with the same dimensions (ca. 12 × 9 cm) as natural grapevine leaves with black cotton (0.5 cm wide) and placed in the own vine canopies (Fig. 1c, d). For wet references water-absorbent non-woven polyester was covered with black cotton and fully soaked immediately prior to the UAV flight. Temperature extraction from these references was corrected by emissivity set to 0.95 and performed manually to ensure pure pixel information from the center of targets. These “references” were used to estimate the lower and upper boundary temperatures corresponding to a fully non-transpiring leaf and transpiring leaf [14].

Data from the meteorological station were employed for *g*_*c*_ (as the leaf conductance of the top canopy leaves that are observed from the UAV) estimation using leaf energy balance budget [15, 16] with the meteorological data previously described in the “Experimental site and environmental conditions” section. The basic leaf energy balance equation (Eq. 4):

4 $$T_{c} - T_{a} = {{ \left[ {r_{HR} \left( {r_{aw} + r_{c} } \right)\gamma R_{ni} - pc_{p} r_{HR} D} \right]} \mathord{\left/ {\vphantom {{ \left[ {r_{HR} \left( {r_{aw} + r_{c} } \right)\gamma R_{ni} - pc_{p} r_{HR} D} \right]} {\left\{ {pc_{p} \left[ {\gamma \left( {r_{aw} + r_{c} } \right) + sr_{HR} } \right]} \right\}}}} \right. \kern-0pt} {\left\{ {pc_{p} \left[ {\gamma \left( {r_{aw} + r_{c} } \right) + sr_{HR} } \right]} \right\}}}$$

where *T*_*c*_ and *T*_*a*_ are the canopy and air temperature (°C), *r*_*HR*_ is the parallel resistance to heat and radiative transfer (s m^−1^), *r*_*aw*_ is the boundary layer resistance to water vapour (s m^−1^), *r*_*c*_ is the canopy resistance to water vapour (this resistance is assumed to be mainly dominated by stomata exchange), *γ* is the psychrometric constant (Pa K^−1^), *R*_*ni*_ is the net isothermal radiation, *p* is the density of air (kg m^−3^), *c*_*p*_ is the specific heat capacity of air (J kg^−1^ K^−1^), *D* is the vapour pressure deficit (Pa) and *s* is the slope of the curve of water vapour pressure related to temperature (Pa °C^−1^). Canopy conductance, *g*_*c*_ (*g*_*c*_ = 1/*r*_*c*_), can be calculated by rearrangement of the Eq. 5 following the methodology proposed previously [5]:

5 $$r_{c} = {{ - pc_{p} r_{HR} \left[ {s\left( {T_{c} - T_{a} } \right) + D} \right]} \mathord{\left/ {\vphantom {{ - pc_{p} r_{HR} \left[ {s\left( {T_{c} - T_{a} } \right) + D} \right]} {\left\{ {\gamma \left[ {\left( {T_{c} - T_{a} } \right)pc_{p} - r_{HR} R_{ni} } \right]} \right\} - r_{aw} }}} \right. \kern-0pt} {\left\{ {\gamma \left[ {\left( {T_{c} - T_{a} } \right)pc_{p} - r_{HR} R_{ni} } \right]} \right\} - r_{aw} }}$$

### Metabolite analysis

Samples from the same leaves that gas-exchange was evaluated were collected at midday in parallel with the UAV aerial campaign and immediately frozen in liquid nitrogen and stored at − 80 °C. Grinding also was performed under frozen conditions using liquid nitrogen. Grapevine leaf tissue (ca. 50 mg) was extracted in 1.4 ml of 100% methanol following the previous methodology [57]. Gas chromatography-time of flight-mass spectrometry (GC-TOFMS) analyses was carried out exactly following published methodological protocols [58]. Profiling of secondary metabolite by LC–MS was performed in negative/positive ion detection mode [59], metabolites were identified by co-elution profile of standard chemicals (quercetin-3-*O*-rutinoside, quercetin-3-*O*-galactoside, quercetin-3-*O*-glucoside, kaempferol-3-*O*-rutinoside, kaempferol-3-*O*-glucoside, myricetin-3-*O*-glucoside) and annotated by MS–MS profile with metabolite databases [59]. Metabolites were finally referenced in a dry matter basis per each plant and treatment. Metadata information about the parameters employed for the GC–MS and LC–MS annotation as well an overview of the metabolite reporting list is shown in the Additional file 2: Table S2.

### Statistics and modelling

Statistics were performed using R [60]. Differences between treatments were tested with one-way ANOVA; in the case of significant differences detected, inter-groups differences (post hoc) were performed using Tukey HSD simple hypothesis testing (p < 0.05), both functions included in the R basic package. Principal component analysis was conducted using the R package “pcaMethods” version 1.48.0 that can be downloaded at: http://www.bioconductor.org/packages/release/bioc/html/pcaMethods.html.

For the partial least squares regression, the profile of each metabolite was given by the combination of the replicates from the three treatments. The (few) missing values were imputed by using a recent random forest imputation method which outperforms other existing alternatives [60–63]. For the PLS regression we used the *pls* package in R environment with the add-on function implementing the variable importance in projection (VIP) for single-response orthogonal scores *plsr* models [64].

## Additional files

**Additional file 1: Figure S1.** Relationships obtained from T_c_ − T_a_, CWSI, IG and I3 thermal indices from the aerial thermographic images and the *g*_*s*_ measured at leaf level (a, b, c and d, *n* = 18) and the stem sap flow (e, f, g and h, *n* = 10) (p < 0.05) at noon 29th August 2012 per each of the irrigation treatments. **Figure S2**. Box-plot of the temperature obtained in the three irrigation treatments by **a** leaf temperature measured in the standard chamber of the infrared gas-analyzer LICOR 6400XT (USA) with a flow of 300 μmol air s^−1^ and with **b** temperature obtained from the thermal camera GOBI384 (Xenics, Belgium) equipped in the UAV multi-copter flying over the vineyard at 15 m height. Data were collected in parallel at noon 29th August 2012. No statistical differences were found between them by ANOVA (p < 0.05). **Figure S3**. Changes in the root mean squared error of prediction (RMSEP) with the number of components employed for the PLS modelling: **a** models for *g*_*c*_ and **b** models for *g*_*s*_. The dashed and full lines denote the lowest and highest RMSEP from the cross-validation. **Figure S4**. Changes in the coefficient of determination (R2) with the number of components employed for the PLS modelling: **a** models for *g*_*c*_ and **b** models for *g*_*s*_. **Figure S5**. Values of the coefficients for the metabolites, used as predictors, in the PLS models with number of components corresponding to the lowest RMSEP: **a** model for *g*_*c*_ and **b** model for *g*_*s*_.

**Additional file 2.** Primary and secondary metabolite reporting list and metadata overview for the processed and annotated samplesemploying GC-MS and LC-MS technologies.

## References

[1] Gago J, Douthe C, Florez-Sarasa I, Escalona JM, Galmes J, Fernie AR, Flexas J, Medrano H (2014). Opportunities for improving leaf water use efficiency under climate change conditions. Plant Sci.

[2] Araus JL, Cairns JE (2014). Field high-throughput phenotyping: the new crop breeding frontier. Trends Plant Sci.

[3] Medrano H, Escalona J, Cifre J, Bota J, Flexas J (2003). A ten-year study on the physiology of two Spanish grapevine cultivars under field conditions: effects of water availability from leaf photosynthesis to grape yield and quality. Funct Plant Biol.

[4] Fernie AR (2012). Grand challenges in plant systems biology: closing the circle(s). Front Plant Sci.

[5] Berni JAJ, Zarco-Tejada PJ, Sepulcre-Cantó G, Fereres E, Villalobos F (2009). Mapping canopy conductance and CWSI in olive orchards using high resolution thermal remote sensing imagery. Remote Sens Environ.

[6] Zarco-Tejada PJ, González-Dugo V, Berni JAJ (2012). Fluorescence, temperature and narrow-band indices acquired from a UAV platform for water stress detection using a micro-hyperspectral imager and a thermal camera. Remote Sens Environ.

[7] Gonzalez-Dugo V, Zarco-Tejada P, Nicolás E, Norte PA, Alarcón JJ, Intrigliolo DS, Fereres E (2013). Using high resolution UAV thermal imagery to assess the variability in the water status of five fruit tree species within a commercial orchard. Precis Agric.

[8] Gonzalez-Dugo V, Zarco-Tejada P, Berni JAJ, Suárez L, Goldhammer D, Fereres E (2012). Almond tree canopy temperature reveals intra-crown variability that is water stress-dependent. Agric For Meteorol.

[9] Obata T, Fernie AR (2012). The use of metabolomics to dissect plant responses to abiotic stresses. Cell Mol Life Sci.

[10] Tohge T, Watanabe M, Hoefgen R, Fernie AR (2013). The evolution of phenylpropanoid metabolism in the green lineage. Crit Rev Biochem Mol Biol.

[11] Brunetti C, George RM, Tattini M, Field K, Davey MP (2013). Metabolomics in plant environmental physiology. J Exp Bot.

[12] Hochberg U, Degu A, Cramer GR, Rachmilevitch S, Fait A (2015). Cultivar specific metabolic changes in grapevines berry skins in relation to deficit irrigation and hydraulic behavior. Plant Physiol Biochem.

[13] Verslues PE, Juenger TE (2011). Drought, metabolites, and Arabidopsis natural variation: a promising combination for understanding adaptation to water-limited environments. Curr Opin Plant Biol.

[14] Idso SB, Jackson RD, Pinter PJ, Reginato RJ, Hatfield JL (1981). Normalizing the stress-degree-day parameter for environmental variability. Agric Meteorol.

[15] Jones HG (1999). Use of infrared thermometry for estimation of stomatal conductance as a possible aid to irrigation scheduling. Agric For Meteorol.

[16] Monteith J, Unsworth M (2007). Principles of environmental physics.

[17] Fiorani F, Schurr U (2013). Future scenarios for plant phenotyping. Annu Rev Plant Biol.

[18] Carreno-Quintero N, Bouwmeester HJ, Keurentjes JJB (2013). Genetic analysis of metabolome-phenotype interactions: from model to crop species. Trends Genet.

[19] Turner D, Lucieer A. Development of an unmanned aerial vehicle (UAV) for hyper resolution vineyard mapping based on visible, multispectral, and thermal imagery. In Proceedings of 34th international symposium on remote sensing of environment, 2011; p. 4.

[20] Gago J, Douthe C, Coopman RE, Gallego PP, Ribas-Carbo M, Flexas J, Escalona J, Medrano H (2015). UAVs challenge to assess water stress for sustainable agriculture. Agric Water Manag.

[21] Gómez-Candón D, Virlet N, Labbé S, Jolivot A, Regnard JL (2016). Field phenotyping of water stress at tree scale by UAV-sensed imagery: new insights for thermal acquisition and calibration. Prec Agric.

[22] Santesteban LG, Di Gennaro SF, Herrero-Langreo A, Miranda C, Royo JB, Matese A (2017). High-resolution UAV-based thermal imaging to estimate the instantaneous and seasonal variability of plant water status within a vineyard. Agric Water Manag.

[23] Berni JAJ, Member S, Zarco-Tejada PJ, Suárez L, Fereres E (2009). Thermal and narrowband multispectral remote sensing for vegetation monitoring from an unmanned aerial vehicle. Remote Sens Environ.

[24] Marino G, Pallozzi E, Cocozza C, Tognetti R, Giovannelli A, Cantini C, Centritto M (2013). Assessing gas exchange, sap flow and water relations using tree canopy spectral reflectance indices in irrigated and rainfed *Olea europaea* L. Environ Exp Bot.

[25] Warren CR, Aranda I, Cano FJ (2011). Responses to water stress of gas exchange and metabolites in Eucalyptus and Acacia spp. Plant Cell Environ.

[26] Witt S, Galicia L, Lisec J, Cairns J, Tiessen A, Araus JL, Palacios-Rojas N, Fernie AR (2012). Metabolic and phenotypic responses of greenhouse-grown maize hybrids to experimentally controlled drought stress. Mol Plant.

[27] Aranjuelo I, Tcherkez G, Molero G, Gilard F, Avice JC, Nogués S (2013). Concerted changes in N and C primary metabolism in alfalfa (*Medicago sativa*) under water restriction. J Exp Bot.

[28] Sulpice R, Nikoloski Z, Tschoep H, Antonio C, Kleessen S, Larhlimi A, Selbig J, Ishihara H, Gibon Y, Fernie AR, Stitt M (2013). Impact of the carbon and nitrogen supply on relationships and connectivity between metabolism and biomass in a broad panel of Arabidopsis accessions. Plant Physiol.

[29] Alseekh S, Tohge T, Wendenberg R, Scossa F, Omranian N, Li J, Kleessen S, Giavalisco P, Pleban T, Mueller-Roeber B, Zamir D, Nikoloski Z, Fernie AR (2015). Identification and mode of inheritance of quantitative trait loci for secondary metabolite abundance in tomato. Plant Cell.

[30] Watkins JM, Hechler PJ, Muday GK (2014). Ethylene-induced flavonol accumulation in guard cells suppresses reactive oxygen species and moderates stomatal aperture. Plant Physiol.

[31] Rizhsky L, Liang H, Shuman J, Shulaev V, Davletova S, Mittler R (2004). When defense pathways collide, the response of Arabidopsis to a combination of drought and heat stress. Plant Physiol.

[32] Harb J, Alseekh S, Tohge T, Fernie AR (2015). Profiling of primary metabolites and flavonols in leaves of two table grape varieties collected from semiarid and temperate regions. Phytochemistry.

[33] Hochberg U, Degu A, Toubiana D, Gendler T, Nikoloski Z, Rachmilevitch S, Fait A (2013). Metabolite profiling and network analysis reveal coordinated changes in grapevine water stress response. BMC Plant Biol.

[34] Savoi S, Wong DC, Arapitsas P, Miculan M, Bucchetti B, Peterlunger E, Fait A, Mattivi F, Castellarin SD (2016). Transcriptome and metabolite profiling reveals that prolonged drought modulates the phenylpropanoid and terpenoid pathway in white grapes (*Vitis vinifera* L.). BMC Plant Biol.

[35] Lawson T, Lefebvre S, Baker NR, Morison JI, Raines CA (2008). Reductions in mesophyll and guard cell photosynthesis impact on the control of stomatal responses to light and CO_2_. J Exp Bot.

[36] Araújo WL, Nunes-Nesi A, Nikoloski Z, Sweetlove LJ, Fernie AR (2012). Metabolic control and regulation of the tricarboxylic acid cycle in photosynthetic and heterotrophic plant tissues. Plant Cell Environ.

[37] Kelly G, David-Schwartz R, Sade N, Moshelion M, Levi A, Alchanatis V, Granot D (2012). The pitfalls of transgenic selection and new roles of AtHXK1: a high level of AtHXK1 expression uncouples hexokinase1-dependent sugar signaling form. Plant Physiol.

[38] Lu P, Zhang SQ, Outlaw WH, Riddle KA (1995). Sucrose: a solute that accumulates in the guard-cell apoplast and guard-cell symplast of open stomata. FEBS Lett.

[39] Kang Y, Outlaw WH, Andersen PC, Fiore GB (2007). Guard-cell apoplastic sucrose concentration—a link between leaf photosynthesis and stomatal aperture size in the apoplastic phloem loader *Vicia faba* L. Plant Cell Environ.

[40] Ritte G, Rosenfeld J, Rohrig K, Raschke K (1999). Rates of sugar uptake by guard cell protoplasts of *Pisum sativum* L. related to the solute requirement for stomatal opening. Plant Physiol.

[41] Kelly G, Moshelion M, David-Schwartz R, Halperin O, Wallach R, Attia Z, Belausov E, Granot D (2013). Hexokinase mediates stomatal closure. Plant J.

[42] Fernie AR, Martinoia E (2009). Malate: Jack of all trades or master of a few?. Phytochemistry.

[43] Araújo W, Nunes-Nesi A, Osorio S, Usadel B, Fuentes D, Nagy R, Balbo I, Lehmann M, Studart-Witkowski C, Tohge T, Martinoia E, Jordana X, DaMatta FM, Fernie AR (2011). Antisense inhibition of the iron-sulphur subunit of succinate dehydrogenase enhances photosynthesis and growth in tomato via an organic acid-mediated effect on stomatal aperture. Plant Cell.

[44] Flexas J, Escalona JM, Evain S, Gulías J, Moya I, Osmond CB, Medrano H (2002). Steady-state chlorophyll fluorescence (Fs) measurements as a tool to follow variations of net CO2 assimilation and stomatal conductance during water-stress in C3 plants. Physiol Plant.

[45] Carriquí M, Cabrera HM, Conesa MÀ, Coopman RE, Douthe C, Gago J, Gallé A, Galmés J, Ribas-Carbo M, Tomás M, Flexas J (2015). Diffusional limitations explain the lower photosynthetic capacity of ferns as compared with angiosperms in a common garden study. Plant Cell Environ.

[46] Tosens T, Nishida K, Gago J, Coopman RE, Cabrera HM, Carriquí M, Laanisto L, Morales L, Nadal M, Rojas R, Talts E, Tomas M, Hanba Y, Niinemets Ü, Flexas J (2016). The photosynthetic capacity in 35 ferns and fern allies: mesophyll CO_2_ diffusion as a key trait. New Phytol.

[47] Medrano H, Tomás M, Martorell S, Escalona JM, Pou A, Fuentes S, Flexas J, Bota J (2014). Improving water use efficiency of vineyards in semi-arid regions. A review. Agron Sustain Dev.

[48] Flexas J, Escalona JM, Medrano H (1998). Down-regulation of photosynthesis by drought under field conditions in grapevine leaves. Funct Plant Biol.

[49] Escalona J, Flexas J, Medrano H (2002). Drought effects on water flow, photosynthesis and growth of potted grapevines. Vitis.

[50] Sakuratani T (1981). A heat balance method for measuring water flux in the stem of intact plants. J Agr Meterol.

[51] Baker J, Bavel C (1987). Measurement of mass flow of water in the stems of herbaceous plants. Plant Cell Environ.

[52] Jones HG (2004). Irrigation scheduling: advantages and pitfalls of plant-based methods. J Exp Bot.

[53] Albertz J. Einfuhrung in die Fernerkundung: Grundlagen der Interpretation von Luft- und Satellitenbildern. 2001.

[54] Lowe D (2004). Distinctive image features from scale-invariant keypoints. Int J Comput Vis.

[55] Snavely N, Seitz SM, Szeliski R (2006). Photo tourism. ACM Trans Graph.

[56] Snavely N, Seitz SM, Szeliski R (2007). Modeling the world from internet photo collections. Int J Comput Vis.

[57] Roessner U, Luedemann A, Brust D, Fiehn O, Linke T, Willmitzer L, Fernie AR (2001). Metabolic profiling allows comprehensive phenotyping of genetically or environmentally modified plant systems. Plant Cell..

[58] Lisec J, Schauer N, Kopka J, Willmitzer L, Fernie AR (2006). Gas chromatography mass spectrometry-based metabolite profiling in plants. Nat Protoc.

[59] Tohge T, Fernie AR (2010). Combining genetic diversity, informatics and metabolomics to facilitate annotation of plant gene function. Nat Protoc.

[60] Team R. R: a language and environment for statistical computing (R Foundation for Statistical Computing, Vienna, 2012). http://www.R-project.org.

[61] Stekhoven DJ, Bühlmann P (2012). Missforest-non-parametric missing value imputation for mixed-type data. Bioinformatics.

[62] Waljee AK, Mukherjee A, Singal AG, Zhang Y, Warren J, Balis U, Marrero J, Zhu J, Higgins PDR (2013). Comparison of imputation methods for missing laboratory data in medicine. BMJ Open.

[63] Gromski PS, Xu Y, Kotze HL, Correa E, Ellis DI, Armitage EG, Turner ML, Goodacre R (2014). Influence of missing values substitutes on multivariate analysis of metabolomics data. Metabolites.

[64] Wehrens R, Mevik BH (2007). The pls Package: Principal Component and Partial Least Squares Regression in R. J Stat Soft..

