# Supplementary material for: Integrative field scale phenotyping for investigating metabolic components of water stress within a vineyard
Source: Plant Methods. 2017 Oct 30;13:90. doi: 10.1186/s13007-017-0241-z (PMC5663058; doi:10.1186/s13007-017-0241-z)

**Integrative field scale phenotyping for investigating metabolic components of water stress within a Mediterranean vineyard**

Jorge Gago, Alisdair R. Fernie, Zoran Nikoloski, Takayuki Tohge, Sebastiá Martorell, José Mariano Escalona, Miquel Ribas-Carbó, Jaume Flexas, Hipólito Medrano

**SUPPLEMENTAL DATA LEGENDS**

Fig. S1. Relationships obtained from T_c_-T_a_, CWSI, IG and I3 thermal indices from the aerial thermographic images and the g_s_ measured at leaf level (a, b, c and d, n=18) and the stem sap flow (e, f, g and h, n=10) (p<0.05) at noon 29^th^ August 2012 per each of the irrigation treatments.

Fig. S2. Box-plot of the temperature obtained in the three irrigation treatments by a) leaf temperature measured in the standard chamber of the infrared gas-analyzer LICOR 6400XT (USA) with a flow of 300 μmol air s^-1^ and with b) temperature obtained from the thermal camera GOBI384 (Xenics, Belgium) equipped in the UAV multi-copter flying over the vineyard at 15 m height. Data were collected in parallel at noon 29^th^ August 2012. No statistical differences were found between them by ANOVA (p<0.05).

Fig. S3. Changes in the root mean squared error of prediction (RMSEP) with the number of components employed for the PLS modelling: (a) models for *g_c_* and (b) models for *g_s_*. The dashed and full lines denote the lowest and highest RMSEP from the cross-validation.

Fig. S4. Changes in the coefficient of determination (R^2^) with the number of components employed for the PLS modelling: (a) models for *g_c_* and (b) models for *g_s_*.

Fig. S5. Values of the coefficients for the metabolites, used as predictors, in the PLS models with number of components corresponding to the lowest RMSEP: (a) model for *g_c_* and (b) model for *g_s_*.

Supplementary Table 1. Relative metabolite content of the fully expanded leaves from the three irrigation treatments. Leaves were harvested at noon in parallel with the UAV flight. Relative log^2^ ratio changes of the treatments (D= drought and C= cover-crop) respect to the mean response of the control watered treatment (W). Different letters means statistical differences by Tukey´s test (p<0.05).

|  | **Treatment** | **W** | **D** | **C** |
| --- | --- | --- | --- | --- |
| **Aminoacids** | **Valine** | 1,00a | 1,60b | 1,04a |
|  | **Isoleucine** | 1,00a | 4,80b | 2,42c |
|  | **Glycine** | 1,00a | 2,03b | 1,47ab |
|  | **Proline** | 1,00a | 1,37b | 0,56c |
|  | **Alanine** | 1,00 ns | 0,93 ns | 0,64 ns |
|  | **Serine** | 1,00 ns | 1,03 ns | 0,89 ns |
|  | **Threonine** | 1,00a | 2,23b | 1,76b |
|  | **Serine O acetyl** | 1,00a | 2,38b | 1,69c |
|  | **Ornithine** | 1,00a | 2,91b | 2,13b |
|  | **Phenylananine** | 1,00a | 1,85b | 1,57ab |
|  | **Asparagine** | 1,00 ns | 2,06 ns | 2,50 ns |
|  | **Glutamine** | 1,00a | 5,94b | 1,84ª |
|  | **Lysine** | 1,00 ns | 1,61 ns | 1,46 ns |
|  | **Tyrosine, DL** | 1,00a | 7,29b | 3,67a |
|  | **Tryptophan** | 1,00a | 4,58b | 2,29a |
| **Organic acids** | **Pyruvic acid** | 1,00 ns | 0,77 ns | 1,16 ns |
|  | **Phosphoric acid** | 1,00a | 0,74b | 0,39b |
|  | **Glyceric acid** | 1,00 ns | 1,17 ns | 1,77 ns |
|  | **Succinic acid** | 1,00 ns | 0,93 ns | 1,00 ns |
|  | **Fumaric acid** | 1,00 ns | 0,89 ns | 0,96 ns |
|  | **Maleic acid** | 1,00a | 0,99a | 1,85b |
|  | **Glutaric acid** | 1,00a | 1,48a | 3,17b |
|  | **Malic acid** | 1,00a | 0,75b | 0,68c |
|  | **Erythronic acid** | 1,00 ns | 1,38 ns | 1,20 ns |
|  | **Butyric acid** | 1,00 ns | 0,95 ns | 0,77 ns |
|  | **Aspartic acid** | 1,00 ns | 1,40 ns | 1,01 ns |
|  | **Threonic acid** | 1,00 ns | 0,91 ns | 0,68 ns |
|  | **Glutamic acid, DL** | 1,00a | 1,32a | 0,63b |
|  | **Citric acid** | 1,00a | 3,83b | 1,17a |
|  | **Isocitric acid** | 1,00a | 3,74b | 1,43a |
|  | **Glyceric acid-3-phosphate** | 1,00 ns | 1,25 ns | 1,04 ns |
|  | **Gulonic acid, 2-oxo-, DL** | 1,00a | 1,29a | 1,74b |
|  | **Galactonic acid-1,4-lactone** | 1,00a | 1,34ab | 1,52b |
|  | **Dehydroascorbic acid dimer** | 1,00 ns | 0,79 ns | 1,15 ns |
|  | **Galactonic acid** | 1,00a | 0,83a | 1,51b |
|  | **Glucuronic acid** | 1,00a | 1,23a | 2,25b |
|  | **Ascorbic acid** | 1,00ab | 2,78b | 0,80a |
|  | **Caffeic acid, trans** | 1,00a | 1,63b | 2,09b |
| **Sugars** | **Ribose** | 1,00a | 0,89b | 0,79c |
|  | **Fructose** | 1,00a | 3,07b | 3,69b |
|  | **Altrose** | 1,00a | 2,03a | 3,25b |
|  | **Glucose** | 1,00a | 2,23b | 3,78b |
|  | **Sucrose** | 1,00ab | 0,85a | 1,16b |
|  | **Maltose** | 1,00 ns | 0,80 ns | 0,88 ns |
|  | **Isomaltose** | 1,00a | 0,83a | 0,49b |
| **Sugar-alcohols** | **Inositol, myo** | 1,00a | 0,51b | 0,51b |
|  | **Galactinol** | 1,00a | 0,99a | 0,35b |
|  | **Glycerol** | 1,00 ns | 0,90 ns | 0,95 ns |
| **Flavonols** | **myricetin-3-O-glactoside** | 1,00 ns | 0,99 ns | 1,07 ns |
|  | **myricetin-3-O-glucoside** | 1,00 ns | 0,88 ns | 0,92 ns |
|  | **myricetin-3-O-glucuronide** | 1,00a | 1,12b | 1,06ab |
|  | **quercetin-3-O-rutinoside** | 1,00 ns | 1,02 ns | 1,02 ns |
|  | **quercetin-3-O-galactoside** | 1,00a | 1,10a | 1,23b |
|  | **quercetin-3-O-glucoside** | 1,00a | 0,98a | 1,10b |
|  | **quercetin-3-O-glucuronide** | 1,00 ns | 1,04 ns | 0,98 ns |
|  | **kaempferol-3-O-rutinoside** | 1,00 ns | 0,97 ns | 1,11 ns |
|  | **kaempferol-3-O-galactoside** | 1,00a | 1,31b | 1,44b |
|  | **kaempferol-3-O-glucoside** | 1,00a | 1,25b | 1,24b |
|  | **kaempferol-3-O-glucuronide** | 1,00 ns | 1,07 ns | 1,12 ns |

Fig. S1

Fig. S2


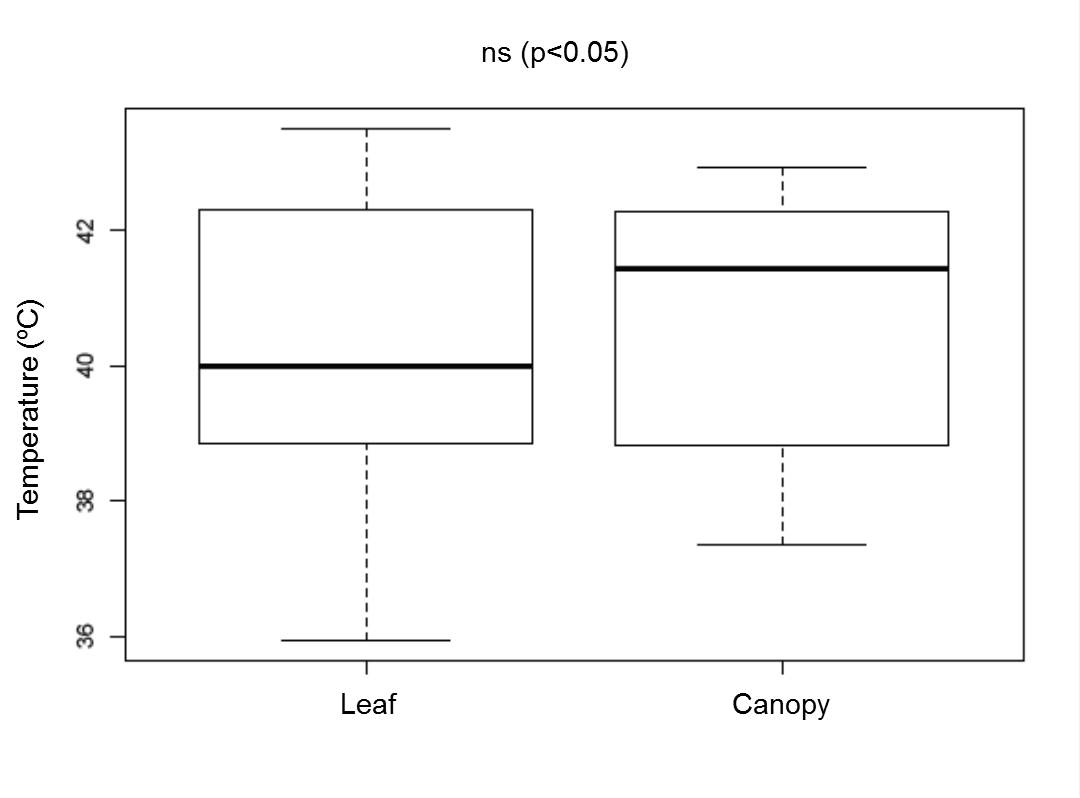


Fig. S3


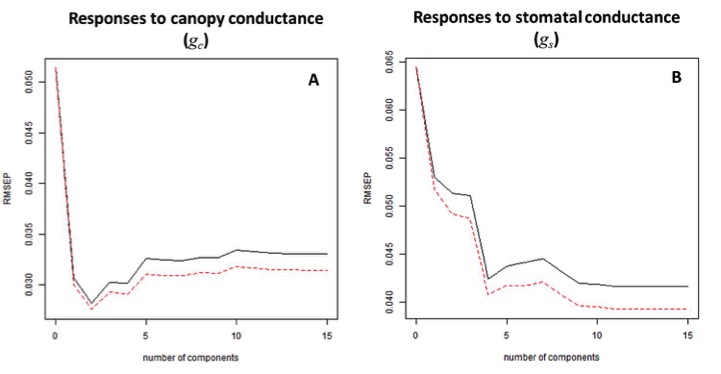


Fig. S4


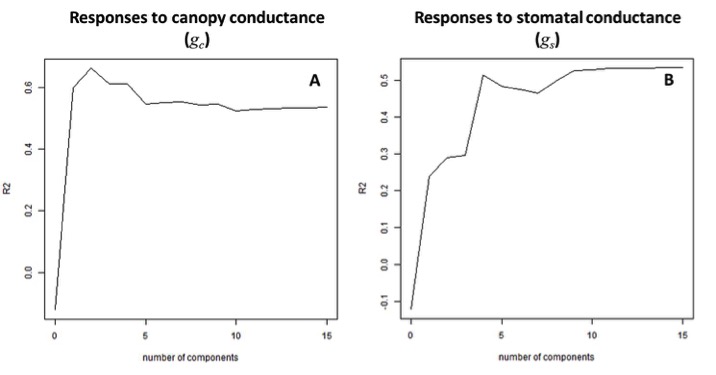


Fig. S5


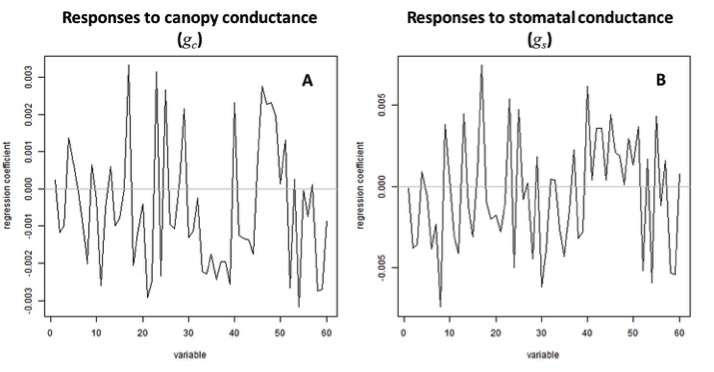

Supplement: Supplementary file 1 — Additional file 1: Figure S1. Relationships obtained from Tc − Ta, CWSI, IG and I3 thermal indices from the aerial thermographic images and the g s measured at leaf level (a, b, c and d, n = 18) and the stem sap flow (e, f, g and h, n = 10) (p < 0.05) at noon 29th August 2012 per each of the irrigation treatments. Figure S2. Box-plot of the temperature obtained in the three irrigation treatments by a leaf temperature measured in the standard chamber of the infrared gas-analyzer LICOR 6400XT (USA) with a flow of 300 μmol air s−1 and with b temperature obtained from the thermal camera GOBI384 (Xenics, Belgium) equipped in the UAV multi-copter flying over the vineyard at 15 m height. Data were collected in parallel at noon 29th August 2012. No statistical differences were found between them by ANOVA (p < 0.05). Figure S3. Changes in the root mean squared error of prediction (RMSEP) with the number of components employed for the PLS modelling: a models for g c and b models for g s. The dashed and full lines denote the lowest and highest RMSEP from the cross-validation. Figure S4. Changes in the coefficient of determination (R2) with the number of components employed for the PLS modelling: a models for g c and b models for g s. Figure S5. Values of the coefficients for the metabolites, used as predictors, in the PLS models with number of components corresponding to the lowest RMSEP: a model for g c and b model for g s. [file 13007_2017_241_MOESM1_ESM.docx]
